# The Nature and Treatment of Deafness, and Diseases of the Ear; and the Treatment of the Deaf and Dumb

**Published:** 1848-12

**Authors:** 


*The Nature and Treatment of Deafness, and Diseases of the Ear; and the Treatment of the Deaf and Dumb.* By WILLIAM DUFTON, M. R. C. S. 12mo. pp. 120. Lea & Blanchard. Philadelphia, 1848.

It is difficult to understand why diseases of the ear have claimed less of the attention of the profession than those of the eye, and of the other important organs; and yet it is probably true. In fact, to a great extent, particularly in America, the diseases of the ear are either left to chance, or the blind counsels of empirics. The organ of hearing is exceedingly complex and delicate in its structure, and consequently liable to many forms of disease which can not be appreciated by any one who is not perfectly familiar with its anatomical character. Tumours may form in it, blocking up its passages, compressing its nerves, or modifying the condition of its circulation; inflammation in one or several of its tissues may and often does occur, followed by the consequences usual in inflammation of the same structures in other parts, and paralysis, more or less complete, of its delicate nerves; and to discover and properly treat these conditions demands equal skill and attention with the complaints of other organs; and yet how seldom do either patients or physicians regard the subject in this serious light. In Europe it is otherwise, particularly in Germany and France; and lately, in England, Mr. Pilcher and Dr. Wharton Jones have made important contributions to our knowledge on this subject.

The little work before us, although quite too brief to afford a complete acquaintance with the subject of aural surgery, is well calculated to awaken attention and beget an interest in the study which may lead to further investigation. The author has treated the diseases of the ear rationally; that is, he has applied the well established principles of pathology to their explanation, and has thus shown that the field is a legitimate one for scientific exploration. To those who have not access to more extended treatises, this manual of Mr. Dufton will afford great assistance in the treatment of aural affections.

